# Effect of photodynamic therapy on choroid of the medial area from optic disc in patients with central serous chorioretinopathy

**DOI:** 10.1371/journal.pone.0282057

**Published:** 2023-02-21

**Authors:** Ryoh Funatsu, Shozo Sonoda, Hiroto Terasaki, Hideki Shiihara, Naohisa Mihara, Juun Horie, Taiji Sakamoto

**Affiliations:** 1 Department of Ophthalmology, Kagoshima University Graduate School of Medical and Dental Sciences, Kagoshima, Japan; 2 Canon Inc., Tokyo, Japan; Massachusetts Eye & Ear Infirmary, Harvard Medical School, UNITED STATES

## Abstract

**Purpose:**

To explore the effect of photodynamic therapy (PDT) on the choroid of medial area from optic disc and factors correlated with treatment outcomes, we evaluated choroidal changes using ultra-widefield optical coherence tomography (UWF-OCT) after PDT for central serous chorioretinopathy (CSC).

**Methods:**

In this retrospective case-series, we included CSC patients who received a standard-dose of full-fluence PDT. UWF-OCT were examined at baseline and 3 months after treatment. We measured choroidal thickness (CT), classified into central, middle, and peripheral sectors. We examined CT changes after PDT by sectors and treatment outcome.

**Results:**

Twenty-two eyes of 21 patients (20 males; mean age 58.7 ± 12.3 years) were included. CT reduction after PDT was significant in all sectors, including peripheral areas: supratemporal, 330.5 ± 90.6 μm vs. 237.0 ± 53.2 μm; infratemporal, 240.0 ± 89.4 μm vs. 209.9 ± 55.1 μm; supranasal, 237.7 ± 59.8 vs 209.3 ± 69.3 μm; infranasal, 172.6 ± 47.2 μm vs. 155.1 ± 38.2 μm (P < 0.001, for all). In patients with retinal fluid resolution, despite no apparent difference in baseline CT, there was more significant reduction after PDT in supratemporal and supranasal peripheral sectors, compared with patients without resolution: supratemporal, 41.9 ± 30.3 μm vs. −1.6 ± 22.7 μm; supranasal, 24.7 ± 15.3 μm vs. 8.5 ± 3.6 μm (P < 0.019, for both).

**Conclusions:**

Whole CT decreased after PDT, including in medial areas from optic disc. This may be associated with the treatment response of PDT for CSC.

## Introduction

Central serous chorioretinopathy (CSC) is a cause of serous retinal detachment and choroidal neovasculopathy (CNV), resulting in severe visual impairment [1–4]. Developments in optical coherence tomography (OCT) and fundus photography [5,6], have revealed different presentations of CSC. Not only does it present with choroidal vascular hyperpermeability (CVH) and choroidal thickening, but it also presents with abnormal patterns of vortex veins [7,8]. These choroidal features are termed *pachychoroid*; CSC is representative of pachychoroid-spectrum disorders. It has been suggested recently that choroidal venous overload is involved in the pathogenesis of pachychoroid-spectrum disorders, and the role of choroid has attracted much attention [9].

The effectiveness of photodynamic therapy (PDT) for CSC has been reported by many studies [2,3,10,11]. PDT decreases choroidal blood flow and choroidal thickness (CT) in the macula; it can affect choroidal circulation and improve disease status in CSC [10,12,13]. Nevertheless, previous studies were limited to analyzing the PDT-irradiated area and its vicinity [10,12–14]; the effect of PDT on peripheral choroid away from the irradiated area is unknown. Above all, medial area from optic disc where blood is supplied by medial short ciliary arteries has not been reported to our knowledge. If choroid of that area is affected by PDT, it indicates the functional association or anastomosis of choroidal circulatory system between medial and lateral area.

The larger area can be observed using ultra-widefield swept-source OCT (UWF-OCT), which has contributed to elucidating the pathogenesis of various retinal diseases [15]. UWF-OCT can capture images from the macula to vortex vein ampulla; it can also observe choroid on the medial side from the optic disc, which has rarely been analyzed before. We believe that we can clarify PDT’s mechanism by studying its effect on the entire choroid. Conventional thickness measurement using OCT is not sufficient as a quantitative method for these areas because of image deformation and distortion that are more prominent at periphery [16]. We developed a method to correct OCT scans to obtain the choroidal images that closely represent the actual shape [17].

We used UWF-OCT to analyze structural changes in the choroid after PDT in CSC patients. We also analyzed differences in choroidal changes by treatment outcomes. This study aimed to investigate PDT’s mechanism and its effect on choroid.

## Materials and methods

### Study design

This single-center, retrospective, observational study was approved by the Ethics Committee of Kagoshima University, Kagoshima, Japan (No. 16012). Although informed consent was not obtained, the study was publicized at the facility and on the website, and the opt-out method ensured that the subjects had the opportunity to refuse participation. All procedures were conducted according to the tenets of the Declaration of Helsinki.

### Subjects

We included consecutive CSC patients who visited the Department of Ophthalmology, Kagoshima University Hospital between April 2021 and March 2022, and who received a standard-dose of verteporfin (6 mg/m^^2^) and full-fluence PDT. All subjects were followed up for 3 months. PDT was performed in patients with serous retinal detachment lasting at least three months at the discretion of the attending physician, and visual acuity was not included in the criteria.

We included only CSC patients with pachychoroid-spectrum disorder features, which we defined as follows based on previous reports: (1) leakage in the serous retinal detachment on fluorescein angiography (FA), (2) dilated Haller’s vessels on OCT B-scan image, en face image, or indocyanine green angiography (ICGA), (3) CVH on ICGA, and (4) no aggregated soft drusen [18–20].

All patients underwent an extensive ophthalmic assessment, including the following: (1) refraction test using an autorefractor (RM8900; Topcon, Tokyo, Japan), (2) best-corrected visual acuity (BCVA) test, (3) intraocular pressure using a computerized tonometer (CT-80; Topcon), (4) axial length using an optical biometer (OA-2000 Optical Biometer; Tomey, Tokyo, Japan), (5) slit-lamp biomicroscopy of the anterior segment and ophthalmoscopy of the ocular fundus, (6) color fundus photography (DRI OCT Triton; Topcon), (7) color scanning laser ophthalmoscopy (California; Optos, Dunfermline, UK), (8) swept-source OCT (DRI OCT Triton), (9) spectral-domain OCT (Spectralis; Heidelberg Engineering, Heidelberg, Germany), (10) UWF-OCT (OCT-S1, Canon, Tokyo, Japan), (11) OCT angiography (PLEX^®^ Elite 9000, ZEISS, Oberkochen, Germany), (12) FA (Mirante; NIDEK, Tokyo, Japan), and (13) ICGA (Mirante). UWF-OCT images were acquired in radial mode. Visual acuity was measured decimally and converted to the logarithm of the minimum angle of resolution (logMAR).

The exclusion criteria were (1) eyes with blurred images; (2) a history of internal eye surgery (except for cataract surgery and laser iridectomy); and (3) eyes with a history of PDT treatment. We also excluded eyes with retinochoroidal disease, except for CSC, glaucoma, and posterior staphyloma. We excluded eyes that did not meet the definition of pachychoroid-spectrum disorders and those diagnosed with secondary CSC due to steroid usage. We further excluded patients with connective tissue disease, current pregnancy, and Cushing’s disease.

### Determination of watershed line of Haller’s layer

The choroidal watershed line was evaluated using 12 × 12 mm OCT en face images (PLEX^®^ Elite 9000). Two retinal specialists (RF and HT) acted as evaluators to classify the watershed line. If their answers were different, a third retinal specialist (SS) made the decision. When the watershed line coincided with the line between fovea and optic disc, the eye was classified as symmetrical; when it was displaced below the fovea–disc line, it was classified as upper predominant, and when it was displaced above the fovea–disc line, it was classified as lower predominant (S1 Fig).

### CT measured with real-shape ultra-widefield OCT

**Fig 1:**
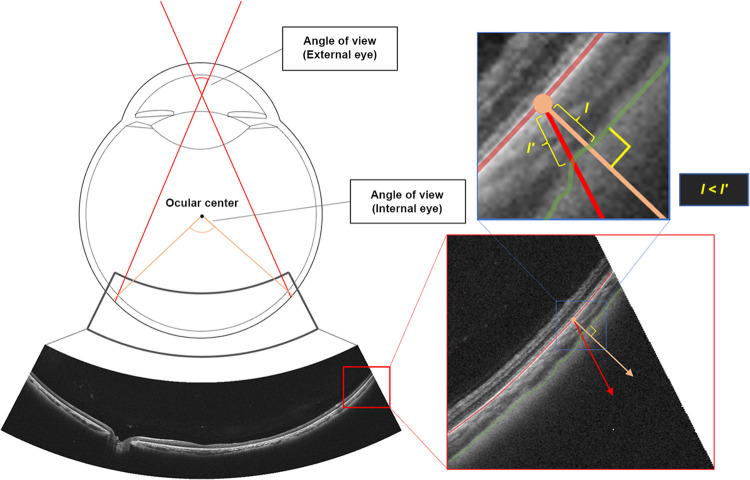
Using UWF-OCT to measure CT. Using UWF-OCT to measure CT thickness, based on light rays incident from the outside of the eye, may overestimate the thickness between the retinal pigment epithelium (red line) and the choroid–sclera boundary (green line) because the measurement is oblique to the fundus (l′). Thus, measuring CT using the center of the eye, which was set on the basis of the measured eye shape, made it possible to measure the thickness more accurately, even with UWF-OCT, because the measurement direction became almost vertical to the fundus (l). **Abbreviations:** UWF-OCT, ultrawide field optical coherence tomography; CT, choroidal thickness.

**Fig 2:**
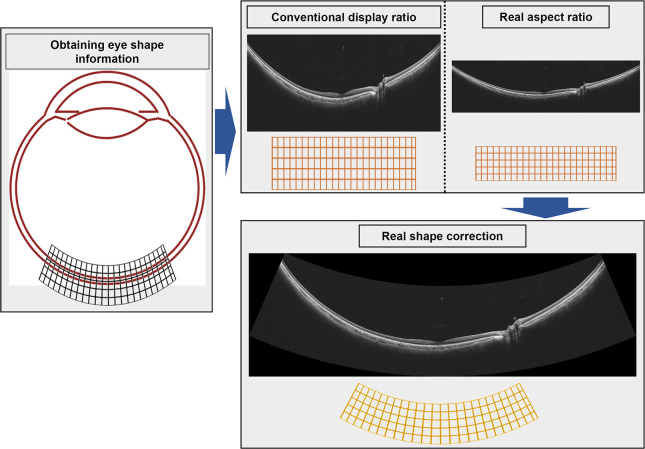
Real-shape correction of OCT images. We acquired information on eye shape when taking ultra-widefield OCT images. Conventional OCT images are displayed stretched vertically, but this method displays them in a 1:1 actual size ratio. Choroidal thickness was analyzed after real-shape correction of the OCT image, to correspond to the eye shape information acquired when the image was taken. **Abbreviations:** OCT, optical coherence tomography.

UWF-OCT images were taken using a UWF-OCT apparatus. Image averaging was set at 20. We used the radial scan mode, centered on the fovea, with an intraocular angle of view of 115° (equivalent to a scan width of 23 mm) However, we considered that the peripheral area was not suitable for analysis because the image was too dark; therefore, we analyzed only the area within an intraocular angle of view of 100° (Fig 1). We manually segmented Bruch’s membrane and the choroid–sclera interface (CSI). Two retina specialists (RF and HT) performed segmentation; if their answers were different, a third retina specialist (SS) made the decision. The eye center was estimated from eye shape information acquired when the images were taken. We defined CT as the difference between Bruch’s membrane and the CSI. Compared with the conventional method of measuring CT, our method allows for better vertical measurement of the retina and choroid, thus reducing deviation in CT measurements via UWF-OCT (Fig 1). In conventional OCT B-scan images, the fundus is displayed and measured in an orthogonal arrangement. Because it is a curved surface, the disparity between measured and actual values becomes more prominent toward the periphery. Thus, to enable more accurate thickness measurements, we used software (EXD Conversion tool for V4.7) provided by Canon to correct OCT images and display them at the true aspect ratio; the images were converted to a real-shape according to the actual eye shape information acquired during scanning **(**Fig 2) [17].

### CT map

**Fig 3:**
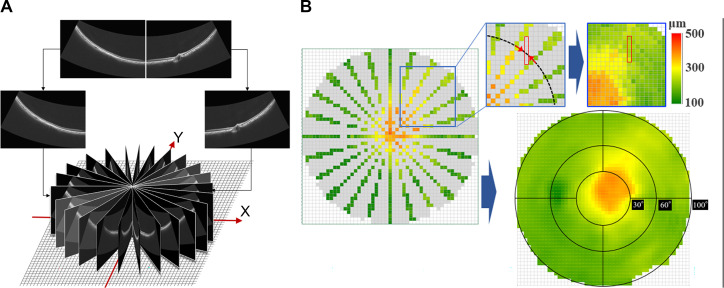
Dividing OCT B scans into sectors. We divided each OCT B image (obtained by 12 radial UWF-OCT scans) into two parts (at the center of the picture). (A) We measured the choroidal thickness at the boundary of the area, divided into 24 parts radially and evenly from the origin on the XY plane. (B) The area between the scans was linearly imputed based on the choroidal thickness measured from two scans in the vicinity of the concentric circles. The distance from the scan lines was measured to create a circular choroidal thickness map, with a diameter of 23 mm. This map was divided into a central area, (between the fovea and 30° from the fovea), a middle area (between 30° and 60° from the fovea), and a peripheral area (between 60° and 100° from the fovea). The middle and peripheral areas were divided into four quadrants: Supratemporal, infratemporal, supranasal, and infranasal. **Abbreviations:** OCT, optical coherence tomography; UWF-OCT, ultra-widefield OCT.

The OCT B-scan images obtained from 12 radial UWF-OCT scans were divided into two sections at the center of the image. CT was measured in 24 evenly divided radial sections from the origin on the X–Y plane (Fig 3A). A CT map was created by linear imputation based on CT, measured from two scans in a concentric neighborhood, and the distance from the scan lines (Fig 3B). The thickness map was divided into three areas: central (from fovea to 30°), middle (30°–60° from fovea), and peripheral (60°–100° from fovea). Furthermore, we horizontally and vertically divided the middle and the peripheral area into four quadrants: supratemporal, infratemporal, supranasal, and infranasal, based on the choroidal watershed (Fig 3B) [21]. Where OCT captured part of optic disc parts or OCT was not clearly captured, it was excluded from the analysis. The mean thickness of each area is reported.

### Outcomes

The primary outcome was the comparison of CT between the subfoveal, central, middle, and peripheral areas at baseline and 3 months after PDT. The secondary outcome was the comparison of patient backgrounds and choroidal thickness before PDT, changes in CT, and complications between cases in which retinal fluid disappeared after treatment and in which retinal fluid remained.

### Statistical analysis

The data are reported as means with standard deviations for continuous variables and as numbers and percentages for qualitative variables. CT in the fovea, central, middle, and peripheral areas were measured at baseline and 3 months after PDT. Using the Wilcoxon signed-rank test, change in the CT was analyzed. We defined “absolute difference” as CT before treatment minus CT after treatment. We defined “decrease proportion” as the absolute difference divided by the CT before treatment, multiplied by 100. Patients were divided into two groups according to the presence of retinal fluid at 3 months after PDT. We used the Mann–Whitney U test for continuous variables and Fisher’s exact test for qualitative variables, to test the differences between the groups in terms of patient background, and CT. We used R software (version 4.0.5) for all analyses. A P-value of 0.05 was used as the cutoff value for significance.

## Results

### Pretreatment characteristics of patients

The subjects were 22 eyes of 21 Japanese patients (age, 58.7 ± 12.3 years; 20 males [90.9%]) with a mean axial length of 23.7 ± 0.9 mm, mean spherical equivalent of −0.16 ± 2.13 diopters, and baseline BCVA of 0.23 ± 0.45 logMAR (Table 1). Five eyes (22.7%) had CNV. Before PDT, SCT was 388.8 ± 93.8 μm and subfoveal retinal thickness was 328.8 ± 117.8 μm. Three eyes (13.6%) were of symmetrical type, 14 eyes (63.6%) of upper predominant type, and five eyes (22.7%) of lower predominant type (Tables 1 and 2). The average interval from the first episode was 35.7 ± 31.4 months.

**Table 1:** Pretreatment characteristics of patients with central serous chorioretinopathy.

Characteristics	No. (%) (n = 22)
Male	20 (90.9%)
Age (years)	58.7 ± 12.3
Left	14 (63.6%)
Axial length (mm)	23.74 ± 0.93
Spherical equivalent (diopter)	-0.16 ± 2.13
BCVA* (logMAR^†^): baseline	0.23 ± 0.45
Asymmetry of Haller’s layer	19 (86.4%)
Running pattern of Haller’s layer	
Symmetry	3 (13.6%)
Upper predominance	14 (63.6%)
Lower predominance	5 (22.7%)
Choroidal hyperpermeability	22 (100%)
Choroidal neovascularization	5 (22.7%)
Treatment history	
None	17 (77.3%)
Photocoagulation	2 (9.1%)
Anti-VEGF^‡^ IV**	3 (13.6%)
Interval from the first episode (months)	35.7 ± 31.4
Classification of central serous chorioretinopathy	
Acute	1 (4.6%)
Chronic	19 (86.4%)
Unknown	2 (9.1%)
Subfoveal retinal thickness (μm)	328.8 ± 117.8
PDT^††^ spot size (μm)	3,984.3 ± 1,224.9

The data are shown as means with standard deviations.

*best-corrected visual acuity; ^†^logarithm of the minimum angle of resolution; ^‡^vascular endothelial growth factor; **intravitreal injection; ^††^photodynamic therapy.

**Table 2:** Changes in choroidal thickness before and after PDT*, by region, in patients with central serous chorioretinopathy.

Characteristics	Baseline (μm)	Three months after PDT (μm)	P-value	The absolute difference (μm)	Decrease proportion (%)
SCT^†^	388.8 ± 93.8	307.6 ± 100.0	<0.001	81.1 ± 68.7	20.4 ± 15.9
Central area, 0°–30°	387.1 ± 78.1	306.7 ± 93.8	<0.001	80.4 ± 63.5	20.8 ± 16.3
Supratemporal, 30°–60°	354.7 ± 80.5	294.7 ± 74.9	<0.001	60.0 ± 44.6	16.5 ± 12.1
Supratemporal, 60°–100°	330.5 ± 90.6	237.0 ± 53.2	<0.001	34.0 ± 33.3	11.5 ± 12.2
Infratemporal, 30°–60°	273.2 ± 69.4	274.4 ± 81.8	<0.001	56.1 ± 41.3	16.4 ± 13.8
Infratemporal, 60°–100°	240.0 ± 89.4	209.9 ± 55.1	<0.001	27.8 ± 29.5	11.0 ± 13.7
Supranasal, 30°–60°	271.0 ± 62.9	237.9 ± 71.2	<0.001	35.3 ± 28.3	13.2 ± 13.2
Supranasal, 60°–100°	237.7 ± 59.8	201.1 ± 61.0	<0.001	21.8 ± 15.2	10.1 ± 7.3
Infranasal, 30°–60°	222.9 ± 62.4	209.3 ± 69.3	0.003	30.7 ± 44.9	10.2 ± 20.4
Infranasal, 60°–100°	172.6 ± 47.2	155.1 ± 38.2	<0.001	17.5 ± 16.1	9.4 ± 7.9

The data are shown as means with standard deviations. P-values were calculated using the Wilcoxon signed-rank test, comparing choroidal thickness before and 3 months after PDT.

*photodynamic therapy; ^†^subfoveal choroidal thickness.

Leakage due to FA and CVH was observed in all patients before PDT. Seventeen eyes (77.3%) had no prior treatment for CSC, two eyes had a history of photocoagulation, and three eyes had received prior intravitreal injection of an anti-vascular endothelial growth factor (VEGF) agent.

### CT change before and after treatment

**Fig 4:**
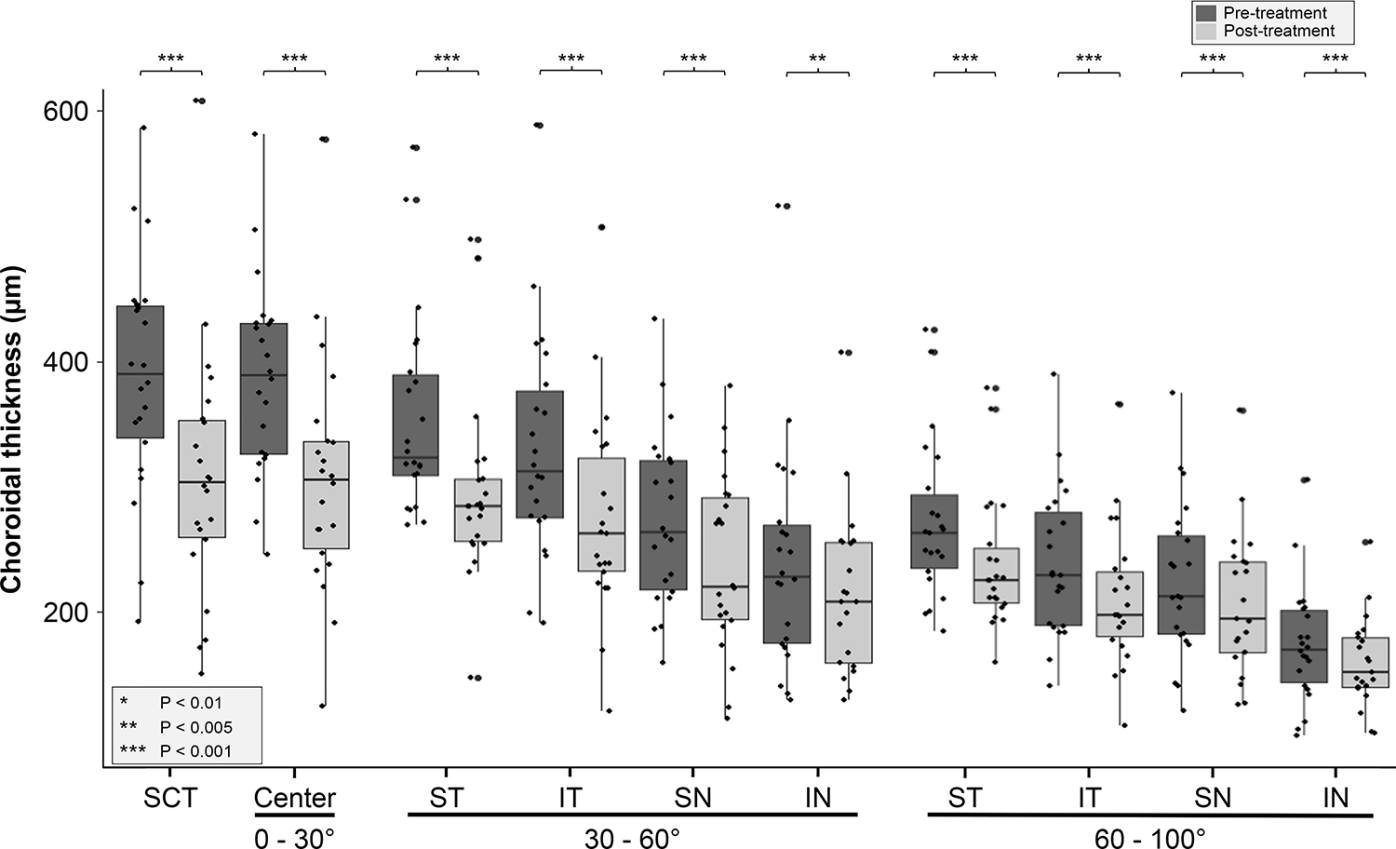
CT changes before and after PDT. Box plots show changes in CT after PDT, for each region. **Abbreviations:** CT, choroidal thickness; PDT, photodynamic therapy; SCT; subfoveal choroidal thickness; ST, supratemporal; IT, infratemporal; SN, supranasal; IN, infranasal.

There was a statistically significant decrease in CT after PDT in all areas. All P-values were ≤ 0.003 (Wilcoxon signed-rank test, Table 2, Fig 4). The absolute differences of CT in the fovea and the central area were 81.1 ± 68.7 μm and 80.4 ± 63.5 μm, respectively; the decrease proportions were 20.4% ± 15.9 and 20.8% ± 16.3, respectively, which were statistically significant (P < 0.001, for both, S2 Fig). A statistically significant reduction of CT was observed in all sectors of the middle area, especially supratemporally. The absolute difference was 60.0 ± 44.6 μm and the decrease proportion was 16.5% ± 12.1, which were the greatest values in the area (P < 0.001). There was a significant reduction in all sectors of the peripheral area, especially supratemporally. The absolute difference was 34.0 ± 33.3 μm and the decrease proportion was 12.4% ± 10.2, which were the greatest values in the area (P < 0.001).

### Differences in ocular characteristics according to treatment outcome

In four eyes (18.2%), retinal fluid did not disappear at 3 months after PDT. As one of the four patients had a persistence of subretinal fluid after PDT, an additional anti-VEGF drug was administered two months after PDT. There was only one case of postoperative hemorrhage in an eye with residual retinal fluid after PDT. There was no difference in the rate of inflammation up to 3 months after PDT [Fluid (−) vs. Fluid (+); 6 (33.3%) vs. 2 (50.0%); P = 0.602] (Table 3).

**Table 3:** Characteristics of patients with central serous chorioretinopathy by treatment outcome.

Characteristics	Subretinal fluid resolution, n = 18	Subretinal fluid persistence, n = 4	P-value
Male	17 (94.4%)	3 (75.0%)	0.338
Age (years)	57.1 ± 12.4	66.3 ± 10.2	0.172
Axial length (mm)	23.78 ± 0.79	23.58 ± 1.57	0.386
Spherical equivalent (D)	−0.41 ± 2.20	1.00 ± 1.49	0.370
BCVA* (logMAR^†^): baseline	0.22 ± 0.47	0.24 ± 0.39	0.764
Subfoveal choroidal thickness	402.56 ± 63.91	326.75 ± 179.41	0.160
Asymmetry of Haller’s layer	16 (88.9%)	3 (75.0%)	0.470
Predominance direction of Haller’s layer			0.744
Symmetry	2 (11.1%)	1. (25.0%)	
Upper predominance	12 (66.7%)	2. (50.0%)	
Lower predominance	4 (22.2%)	1. (25.0%)	
Choroidal neovasculopathy: Baseline	2 (11.1%)	3 (75.7%)	0.024
Treatment history			0.094
None	15 (83.3%)	2 (50.0%)	
Photocoagulation	2 (11.1%)	0 (0.0%)	
Anti-VEGF^‡^ IV**	1 (5.6%)	2 (50.0%)	
Interval from the first episode (months)	35.2 ± 31.9	38.3 ± 34.8	0.791
Classification of central serous chorioretinopathy			0.474
Acute	1 (5.6%)	0 (0%)	
Chronic	16 (88.9%)	3 (75.0%)	
Unknown	1 (5.6%)	1 (25.0%)	
Subfoveal retinal thickness (μm)	321.6 ± 103.9	361.5 ± 185.2	0.999
PDT spot size (μm)	3,924.1 ± 1,212.2	4,255.50 ± 1,433.91	0.652
Complication: Inflammation	6 (33.3%)	2 (50.0%)	0.602
Complication: Hemorrhage	0 (0.0%)	1 (25.0%)	0.182
BCVA (logMAR): After PDT^††^	0.09 ± 0.30	0.20 ± 0.34	0.463

The data are shown as means with standard deviations. P-values were calculated using Fisher’s exact test for ordinal data and the Mann–Whitney U test for continuous data.

*best-corrected visual acuity; ^†^logarithm of the minimum angle of resolution; ^‡^vascular endothelial growth factor; **intravitreal injection; ^††^photodynamic therapy.

Eyes in the residual retinal fluid group were older and had poor visual acuity at 3 months post-treatment. The proportion of CNV cases was significantly higher in eyes with residual retinal fluid group (P = 0.024; Fisher’s exact test). There were no differences in axial length, spherical equivalent, treatment history, or PDT spot size (P ≥ 0.094, for all; Fisher’s exact test, Mann–Whitney U test). Comparisons of CT with a retinal fluid difference at 3 months after PDT showed no apparent difference in all regions (P ≥ 0.160, for all; Mann–Whitney U test, S1 Table).

Table 4 shows a comparison of CT changes, before and 3 months after PDT, with and without residual retinal fluid.

**Table 4:** Changes in choroidal thickness before and after photodynamic therapy in patients with central serous chorioretinopathy by region and treatment outcome.

	Choroidal thickness difference (Baseline–3 months after PDT*)		
Characteristics (μm)	Subretinal fluid resolution, n = 18	Subretinal fluid persistence, n = 4	P-value
SCT^†^	98.3 ± 63.6	4.0 ± 19.8	0.003
Central area, 0°–30°	94.0 ± 62.3	19.3 ± 13.0	0.005
Supratemporal, 30°–60°	70.8 ± 41.8	11.6 ± 16.0	0.001
Supratemporal, 60°–100°	41.9 ± 30.3	−1.6 ± 22.7	0.010
Infratemporal, 30°–60°	61.1 ± 41.6	33.7 ± 36.5	0.217
Infratemporal, 60°–100°	35.3 21.3	−5.5 ± 41.5	0.053
Supranasal, 30°–60°	38.1 ± 28.6	22.52 ± 26.2	0.300
Supranasal, 60°–100°	24.7 ± 15.3	8.5 ± 3.6	0.019
Infranasal, 30°–60°	30.0 ± 43.9	34.0 ± 56.4	0.774
Infranasal, 60°–100°	17.6 ± 13.5	17.3 ± 28.1	0.837

The data are shown as means with standard deviations. P-values were calculated using the Mann–Whitney U test.

*photodynamic therapy; ^†^subfoveal choroidal thickness.

The changes in foveal CT were 98.28 ± 63.64 μm and 4.00 ± 19.78 μm in patients with and without residual fluid, respectively (P = 0.003; Mann–Whitney U test). In the central area, the changes 94.0 ± 62.3 μm and 19.3 ± 13.0 μm, respectively (P = 0.005). The absolute difference was more significant in cases without residual retinal fluid. In the supratemporal sector of the middle area, the mean absolute differences in CT were 70.8 ± 41.8 μm and 11.6 ± 16.0 μm, in eyes with and without residual fluid 3 months after PDT, respectively. There was a more significant reduction in eyes with fluid resolution (P = 0.001). In the supratemporal sector of the peripheral area, the mean absolute differences in CT were 41.9 ± 30.3 μm and −1.6 ± 22.7 μm, in eyes with and without residual fluid 3 months after PDT, respectively. There were more significant reductions in eyes with fluid loss (P = 0.010). In the supranasal sector of the peripheral area, the mean absolute differences in CT were 24.7 ± 15.3 μm and 8.5± 3.6 μm, in eyes with and without residual fluid 3 months after PDT, respectively. The reduction was more significant in eyes with fluid loss (P = 0.019). We did not find any statistically significant differences in any other area (P ≥ 0.053).

## Discussion

PDT has been used for pachychoroid-spectrum disorders [2,22–25]. Although the effects of PDT have been studied, its detailed mechanism is unclear [10,12,22,23,26]. We evaluated the effects of PDT on the entire choroid, including medial area from optic disc and peripheral area, which earlier studies have little examined. We found that PDT affects CT at all of these areas. Furthermore, we found that PDT-induced changes in the peripheral choroid are associated with the treatment outcomes.

In PDT, light exposure activates verteporfin to generate oxidative species that cause platelet aggregation and the release of inflammatory substances, which leads to vascular constriction and occlusion [27]. Pathological studies have demonstrated that PDT can occlude CNV and choriocapillaris without damaging choroidal large vessels [28]. Kumashiro et al. reported that choroidal blood flow decreased in CSC patients after PDT-irradiation [13]. Based on these results, it is reasonable that the decrease in CT in and around the irradiated area can be explained as a result of the reduction of choroidal volume caused by PDT.

Hayreh reported that both arterial and venous choroidal circulation have a watershed zone dividing the perfusion from the vicinity of optic disc to temporal and nasal sides [29]. Nevertheless, this does not fully explain why, in our results, PDT applied to the macula reduced the thickness of the entire choroid. In a study that used electron microscopy to observe choroidal vessel casts from human donors, Yoneya et al. reported that arteries and veins in close proximity form interarterial and intervenous shunts [30]. However, whether shunt-vessels exist, even in the watershed zone, is unclear. Recently, Spaide et al. and Matsumoto et al. have reported anastomosis in the watershed zone in pachychoroid-spectrum disorders; their involvement in pathogenesis has attracted attention [31,32].

The present results showed that PDT decreased the choroidal volume beyond the vertical watershed, which could indicate the presence of anastomosis in the watershed from a functional perspective. A finding that PDT irradiation of the macula reduces choroidal thickness in the upper and lower lateral regions is fully understandable since direct blood supply in each region was reduced by PDT to macula. In contrast, the reduction in choroidal thickness medial to the optic nerve has a different meaning. Suppose that a vertical divide blocks the choroidal circulation on the lateral and medial side. Since the choroid on the nasal side of the optic nerve is supplied by the short posterior ciliary artery, even if PDT reduces the blood supply to the area lateral side of the vertical divide, the effect should be negligible or minimal. However, choroidal thickness of medial side from optic disc was found to be significantly reduced after PDT irradiation to the macula. This suggests that active functional anastomosis is occurring beyond the vertical watershed, and it is further important that the present study clarified this point using a 3 dimensional or functional analysis of choroidal thickness, whereas Spaide and Matsumoto’s report was based on a 2-dimensional analysis using angiography or en-face images [31,32].

By treatment outcome after PDT for CSC, we found a significant difference only in terms of having CNV (P ≥ 0.024). This indicates that the presence of CNV could be a predictor of response to PDT in CSC. It is known that CSC with a chronic course can develop CNV [1]. It is possible that the eyes with CNV had a longer clinical course and were at a stage where the retinal pigment epithelium and choroid were more damaged and less responsive to PDT than the eyes without CNV. Additionally, there are differences in proinflammatory cytokine profiles between acute CSC, chronic CSC, and CSC with CNV; VEGF concentrations tend to be higher in CSC with CNV [33]. Therefore, these differences between CSC, with or without CNV, may have influenced our results.

Concerning patient background and CT, the only difference was the presence or absence of CNV. The absolute differences between the groups with and without retinal fluid at 3 months after PDT treatment were 98.3 ± 63.6 μm and 4.0 ± 19.8 μm, respectively, in the SCT (P = 0.002) and 94.0 ± 62.3 μm and 19.3 ± 13.0 μm, respectively, in the central area (P = 0.008). There was a significant difference in decreased CT in the PDT-irradiated area in eyes with retinal fluid resolution (Table 4). For the middle area of supratemporal sector and the peripheral areas of supratemporal and supranasal sectors, which were outside the PDT-irradiated area, eyes with a good response to PDT showed a significant decrease in CT, compared with eyes that had a poor response to treatment (P ≤ 0.019). In the group for which PDT was effective, there was a more significant CT reduction within and outside the irradiation area.

Several prospective studies have reported the high efficacy of PDT for CSC; however, they reported a certain percentage of patients having poor responses to PDT [2,3,11]. In other words, some CSC have pathologies that can be ameliorated well by PDT, whereas others do not. Our results clearly demonstrate some differences; different choroidal circulation responses to PDT could affect the efficacy of the treatment. There may be subtypes in CSC with pachychoroid features.

One of the strengths of this study is that we could observe peripheral choroid using UWF-OCT. Furthermore, we were able to evaluate CT more accurately by using real-shape correction [17]. Conversely, our study has some limitations. First, it was a single-center, retrospective study with a relatively small number of patients. Our evaluations of Haller’s vessels asymmetry were subjective. Additionally, we used OCT to evaluate changes based on morphological characteristics; blood flow was not quantified using laser speckle fundus flowgraphy [13]. In future research, it would be desirable to combine OCT and laser speckle flowgraphy in a larger sample size; prospective research is desirable. Our results support the possibility of anastomosis in the watershed of pachychoroid-spectrum disorders. Previous studies used only subjective determinations of anastomosis, using OCT en face images or IA, with which it is possible that overlapping vessels can be evaluated as anastomosis [31,32]. Further studies on anastomosis in the watershed are needed. Moreover, as a previous report has indicated that the treatment outcome of CSCs differed by the FA leakage type [34], future studies should examine the relationship between the pattern of FA leakage and the choroidal response to PDT. Finally, since the current study was a case series non-comparative study, we cannot rule out the possibility of choroidal thickness reduction due to the natural course of the condition. The efficacy of PDT for chronic CSC has been reported previously, however [2,3,11], and it is ethically challenging to follow up on chronic CSC without treatment. In addition, since the current results are relatively large compared to choroidal changes during spontaneous remission in acute CSC [35,36], we believe that the choroidal changes observed in this study were related to PDT.

In conclusion, the effect of PDT on CSC is not limited to the irradiated area, reducing the CT in all choroidal regions including the area beyond the vertical watershed zone. Patients who responded poorly to PDT had a smaller CT reduction than those who responded well. This indicates that some CSC conditions may improve with a reduction of blood inflow, whereas others may not.

## Supporting information

S1 Fig Classification of the choroidal watershed zone. 12 × 12 mm OCT en face images showing choroidal watershed zone patterns.
(TIF)

S2 Fig CT after PDT for each region. CT after PDT for each region, in terms of absolute difference (μm) and proportion of decrease proportion (%). The red circle indicates the optic disc. **Abbreviations:** CT, choroidal thickness; PDT, photodynamic therapy; ST, supratemporal; IT, infratemporal; SN, supranasal; IN, infranasal.
(TIF)

S1 Table Pretreatment choroidal thickness of patients with central serous chorioretinopathy by treatment outcome. The data are shown as means with standard deviations. P-values were calculated using the Mann–Whitney U test.
(DOCX)

## References

[1] Shiragami C, Takasago Y, Osaka R, Kobayashi M, Ono A, Yamashita A, et al. Clinical features of central serous chorioretinopathy with Type 1 choroidal neovascularization. Am J Ophthalmol 2018; 193:80–86. doi: 10.1016/j.ajo.2018.06.009 29940168

[2] Bae SH, Heo J, Kim C, Kim TW, Shin JY, Lee JY, et al. Low-fluence photodynamic therapy versus ranibizumab for chronic central serous chorioretinopathy: one-year results of a randomized trial. Ophthalmology 2014; 121:558–565. doi: 10.1016/j.ophtha.2013.09.024 24268858

[3] Zhao M, Zhang F, Chen Y, Dai H, Qu J, Dong C, et al. A 50% vs 30% dose of verteporfin (photodynamic therapy) for acute central serous chorioretinopathy: one-year results of a randomized clinical trial. JAMA Ophthalmol 2015; 133:333–340. doi: 10.1001/jamaophthalmol.2014.5312 25555191

[4] Spaide RF, Campeas L, Haas A, Yannuzzi LA, Fisher YL, Guyer DR, et al. Central serous chorioretinopathy in younger and older adults. Ophthalmology 1996; 103:2070–2079; discussion 2079–2080. doi: 10.1016/s0161-6420(96)30386-2 9003341

[5] Spaide RF, Koizumi H, Pozzoni MC. Enhanced depth imaging spectral-domain optical coherence tomography. Am J Ophthalmol 2008; 146:496–500. doi: 10.1016/j.ajo.2008.05.032 18639219

[6] Srinivasan VJ, Adler DC, Chen Y, Gorczynska I, Huber R, Duker JS, et al. Ultrahigh-speed optical coherence tomography for three-dimensional and en face imaging of the retina and optic nerve head. Invest Ophthalmol Vis Sci 2008; 49:5103–5110. doi: 10.1167/iovs.08-2127 18658089 PMC2743183

[7] Shiihara H, Sonoda S, Terasaki H, Kakiuchi N, Yamashita T, Uchino E, et al. Quantitative analyses of diameter and running pattern of choroidal vessels in central serous chorioretinopathy by en face images. Sci Rep 2020; 10:9591. doi: 10.1038/s41598-020-66858-1 32533066 PMC7293258

[8] Pang CE, Shah VP, Sarraf D, Freund KB. Ultra-widefield imaging with autofluorescence and indocyanine green angiography in central serous chorioretinopathy. Am J Ophthalmol 2014; 158:362–371.e2. doi: 10.1016/j.ajo.2014.04.021 24794091

[9] Spaide RF, Gemmy Cheung CM, Matsumoto H, Kishi S, Boon CJF, van Dijk EHC, et al. Venous overload choroidopathy: a hypothetical framework for central serous chorioretinopathy and allied disorders. Prog Retin Eye Res 2022; 86:100973. doi: 10.1016/j.preteyeres.2021.100973 34029721

[10] Iovino C, Au A, Chhablani J, Parameswarappa DC, Rasheed MA, Cennamo G, et al. Choroidal anatomic alterations after photodynamic therapy for chronic central serous chorioretinopathy: a multicenter study. Am J Ophthalmol 2020; 217:104–113. doi: 10.1016/j.ajo.2020.04.022 32360342

[11] van Dijk EHC, Fauser S, Breukink MB, Blanco-Garavito R, Groenewoud JMM, Keunen JEE, et al. Half-dose photodynamic therapy versus high-density subthreshold micropulse laser treatment in patients with chronic central serous chorioretinopathy: the PLACE trial. Ophthalmology 2018; 125:1547–1555. doi: 10.1016/j.ophtha.2018.04.021 29776672

[12] Manabe S, Shiragami C, Hirooka K, Izumibata S, Tsujikawa A, Shiraga F. Change of regional choroid thickness after reduced-fluence photodynamic therapy for chronic central serous chorioretinopathy. Am J Ophthalmol 2015; 159:644–651. doi: 10.1016/j.ajo.2015.01.006 25595669

[13] Kumashiro S, Takagi S, Itokawa T, Tajima A, Kobayashi T, Hori Y. Decrease in choroidal blood flow after half and one-third dose verteporfin photodynamic therapy for chronic central serous chorioretinopathy. BMC Ophthalmol 2021; 21:241. doi: 10.1186/s12886-021-01980-w 34053440 PMC8165776

[14] Flores-Moreno I, Arcos-Villegas G, Sastre M, Ruiz-Medrano J, Arias-Barquet L, Duker JS, et al. Changes in choriocapillaris, Sattler, and Haller layer thicknesses in central serous chorioretinopathy after half-fluence photodynamic therapy. Retina 2020; 40:2373–2378. doi: 10.1097/IAE.0000000000002764 31985718

[15] Shinohara K, Tanaka N, Jonas JB, Shimada N, Moriyama M, Yoshida T, et al. Ultrawide-field OCT to investigate relationships between myopic macular retinoschisis and posterior staphyloma. Ophthalmology 2018; 125:1575–1586. doi: 10.1016/j.ophtha.2018.03.053 29716783

[16] Izumi T, Maruko I, Kawano T, Sakaihara M, Iida T, Morphological differences of choroid in central serous chorioretinopathy determined by ultra-widefield optical coherence tomography. Graefes Arch Clin Exp Ophthalmol 2022; 260:295–301. doi: 10.1007/s00417-021-05380-0 34410483

[17] Matsumoto K, Tomatsu N. Tomographic imaging apparatus and photographing method. US Patent 2015; 9:181 B2.

[18] Pang CE, Freund KB. Pachychoroid neovasculopathy. Retina 2015; 35:1–9. doi: 10.1097/IAE.0000000000000331 25158945

[19] Matsumoto H, Kishi S, Mukai R, Akiyama H. Remodeling of macular vortex veins in pachychoroid neovasculopathy. Sci Rep 2019; 9:14689. doi: 10.1038/s41598-019-51268-9 31605004 PMC6789012

[20] Siedlecki J, Schworm B, Priglinger SG. The Pachychoroid disease spectrum-and the need for a uniform classification system. Ophthalmol Retina 2019; 3:1013–1015. doi: 10.1016/j.oret.2019.08.002 31810570

[21] Hayreh SS, Baines JA. Occlusion of the vortex veins. An experimental study. Br J Ophthalmol 1973; 57:217–238. doi: 10.1136/bjo.57.4.217 4196493 PMC1214874

[22] Hata M, Tagawa M, Oishi A, Kawashima Y, Nakata I, Akagi-Kurashige Y, et al. Efficacy of photodynamic therapy for polypoidal choroidal vasculopathy associated with and without Pachychoroid phenotypes. Ophthalmol Retina 2019; 3:1016–1025. doi: 10.1016/j.oret.2019.06.013 31606329

[23] Kitajima Y, Maruyama-Inoue M, Ito A, Sato S, Inoue T, Yamane S, et al. One-year outcome of combination therapy with intravitreal anti-vascular endothelial growth factor and photodynamic therapy in patients with pachychoroid neovasculopathy. Graefes Arch Clin Exp Ophthalmol 2020; 258:1279–1285. doi: 10.1007/s00417-020-04661-4 32236705

[24] van Rijssen TJ, van Dijk EHC, Yzer S, Ohno-Matsui K, Keunen JEE, Schlingemann RO, et al. Central serous chorioretinopathy: Towards an evidence-based treatment guideline. Prog Retin Eye Res. 2019 Nov;73:100770. doi: 10.1016/j.preteyeres.2019.07.003 Epub 2019 Jul 15. 31319157

[25] van Rijssen TJ, van Dijk EHC, Tsonaka R, Feenstra HMA, Dijkman G, Peters PJH, et al. Half-Dose Photodynamic Therapy Versus Eplerenone in Chronic Central Serous Chorioretinopathy (SPECTRA): A Randomized Controlled Trial. Am J Ophthalmol. 2022 Jan;233:101–110. doi: 10.1016/j.ajo.2021.06.020 Epub 2021 Jun 29. 34214454

[26] Maruko I, Iida T, Sugano Y, Ojima A, Ogasawara M, Spaid RF, Subfoveal choroidal thickness after treatment of central serous chorioretinopathy. Ophthalmology. 2010 Sep;117(9):1792–9. doi: 10.1016/j.ophtha.2010.01.023 Epub 2010 May 15. 20472289

[27] Henderson BW, Dougherty TJ. How does photodynamic therapy work? Photochem Photobiol 1992; 55:145–157. doi: 10.1111/j.1751-1097.1992.tb04222.x 1603846

[28] Kramer M, Miller JW, Michaud N, Moulton R, Flotte T, Gragoudas E, Liposomal benzoporphyrin derivative verteporfin photodynamic therapy. Selective treatment of choroidal neovascularization in monkeys. Ophthalmology 1996; 103:427–438. doi: 10.1016/s0161-6420(96)30675-1 8600419

[29] Hayreh SS. In vivo choroidal circulation and its watershed zones. Eye (Lond). 1990;4:273–289. doi: 10.1038/eye.1990.39 2199236

[30] Yoneya S, Tso MO. Angioarchitecture of the human choroid. Arch Ophthalmol 1987; 105:681–687. doi: 10.1001/archopht.1987.01060050099046 3619746

[31] Matsumoto H, Hoshino J, Mukai R, Nakamura K, Kikuchi Y, Kishi S, et al. Vortex vein anastomosis at the watershed in Pachychoroid spectrum diseases. Ophthalmol Retina 2020; 4:938–945. doi: 10.1016/j.oret.2020.03.024 32651158

[32] Spaide RF, Ledesma-Gil G, Gemmy Cheung CM. Intervortex venous anastomosis in pachychoroid-related disorders. Retina 2021; 41:997–1004. doi: 10.1097/IAE.0000000000003004 33109938

[33] Terao N, Koizumi H, Kojima K, Yamagishi T, Nagata K, Kitazawa K, et al. Association of upregulated angiogenic cytokines with choroidal abnormalities in chronic central serous chorioretinopathy. Invest Ophthalmol Vis Sci 2018; 59:5924–5931. doi: 10.1167/iovs.18-25517 30551200

[34] van Rijssen TJ, van Dijk EHC, Scholz P, Breukink MB, Blanco-Garavito R, Souied EH, et al. Focal and Diffuse Chronic Central Serous Chorioretinopathy Treated With Half-Dose Photodynamic Therapy or Subthreshold Micropulse Laser: PLACE Trial Report No. 3. Am J Ophthalmol. 2019;205:1–10. doi: 10.1016/j.ajo.2019.03.025 30951686

[35] Ünlü C, Erdogan G, Gezginaslan TA, Akcay BI, Kardes E, Bozkurt TK. Subfoveal choroidal thickness changes after intravitreal bevacizumab therapy for central serous chorioretinopathy. Arq Bras Oftalmol. 2016;79(5):308–311. doi: 10.5935/0004-2749.20160088 27982209

[36] Yumusak E, Gokcinar NB, Ornek K. Choroidal thickness changes in non-treated acute and ranibizumab-treated chronic central serous chorioretinopathy. Medicine (Baltimore). 2018;97(43):e12885. doi: 10.1097/MD.0000000000012885 30412084 PMC6221658

