# Correction: Effect of photodynamic therapy on choroid of the medial area from optic disc in patients with central serous chorioretinopathy

**DOI:** 10.1371/journal.pone.0342442

**Published:** 2026-02-09

**Authors:** The PLOS One Editors

## Notice of Republication

This article [1] was republished on December 22, 2025, to address an issue identified post-publication. An updated version of S1 Data is provided with this notice. Please download this article again to view the correct version.

The article’s Data Availability statement is updated to: All relevant data are within the paper and its Supporting Information files.

## Supporting information

S1 Data (XLSX)
