# Supplementary figures and images for: Effect of photodynamic therapy on choroid of the medial area from optic disc in patients with central serous chorioretinopathy
Source: PLoS One. 2023 Feb 21;18(2):e0282057. doi: 10.1371/journal.pone.0282057 (PMC9942968; doi:10.1371/journal.pone.0282057)

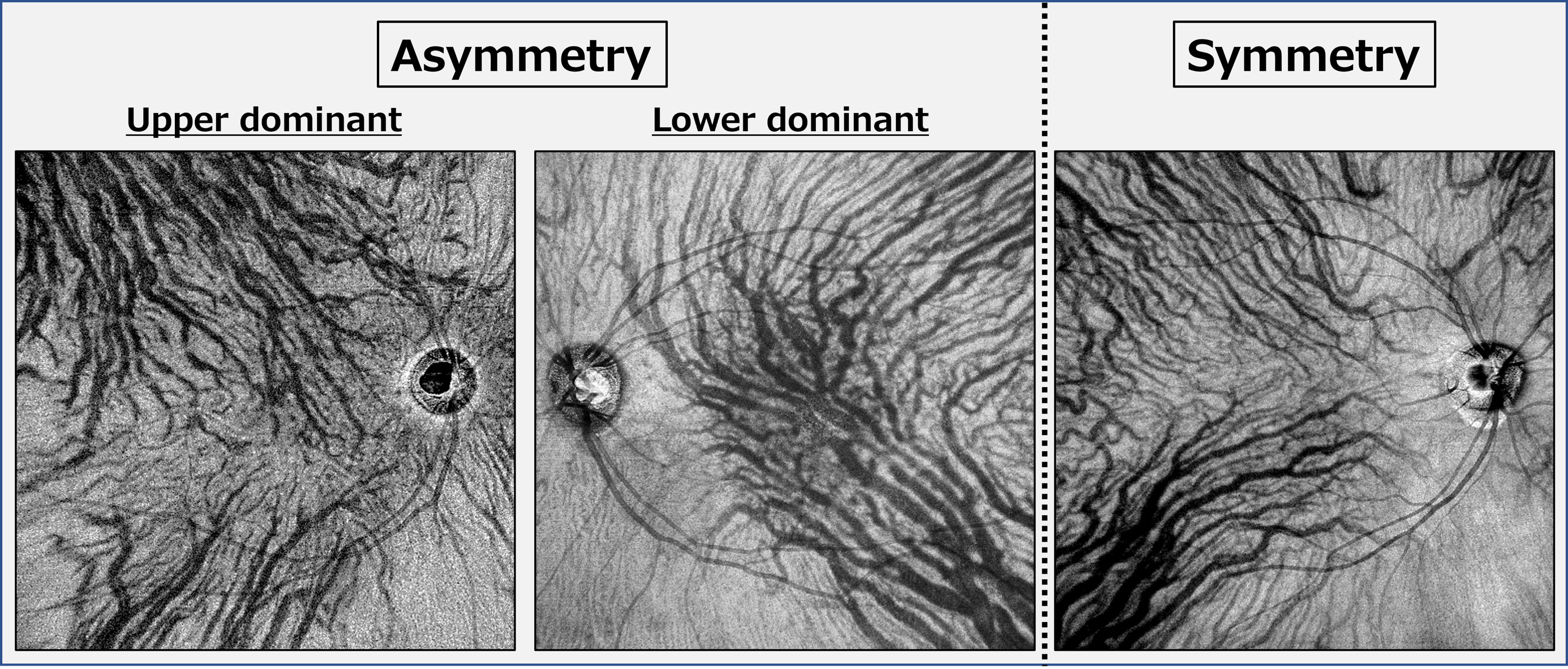

Supplement: S1 Fig — 12 × 12 mm OCT en face images showing choroidal watershed zone patterns. (TIF) [file pone.0282057.s001.tif]

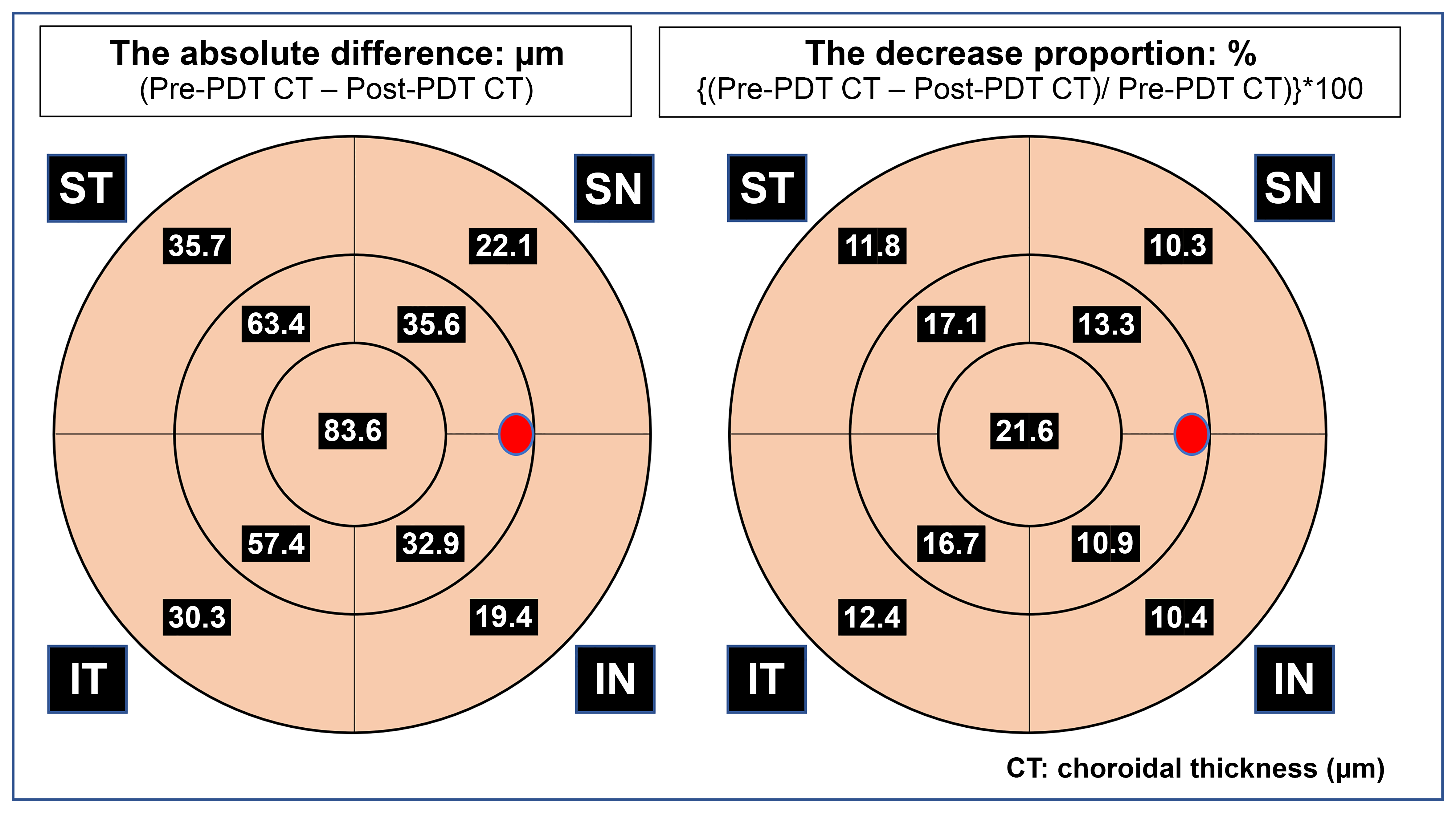

Supplement: S2 Fig — CT after PDT for each region, in terms of absolute difference (μm) and proportion of decrease proportion (%). The red circle indicates the optic disc. Abbreviations: CT, choroidal thickness; PDT, photodynamic therapy; ST, supratemporal; IT, infratemporal; SN, supranasal; IN, infranasal. (TIF) [file pone.0282057.s002.tif]
