# Supplementary material for: Effect of photodynamic therapy on choroid of the medial area from optic disc in patients with central serous chorioretinopathy
Source: PLoS One. 2023 Feb 21;18(2):e0282057. doi: 10.1371/journal.pone.0282057 (PMC9942968; doi:10.1371/journal.pone.0282057)
Supplement: S1 Table — The data are shown as means with standard deviations. P-values were calculated using the Mann–Whitney U test. (DOCX) [file pone.0282057.s003.docx]

**Supplement Table 1.** Pretreatment choroidal thickness of patients with central serous chorioretinopathy by treatment outcome

| Area (μm) | Subretinal fluid resolution, n = 18 | Subretinal fluid persistence, n = 4 | P-value |
| --- | --- | --- | --- |
| Subfoveal choroidal thickness | 402.6 ± 63.9 | 326.8 ± 179.4 | 0.160 |
| Central area, 0°–30° | 394.2 ± 55.2 | 355.2 ± 154.2 | 0.195 |
| Supratemporal, 30°–60° | 357.1 ± 72.4 | 344.2 ± 124.2 | 0.227 |
| Supratemporal, 60°–100° | 273.9 ± 54.7 | 258.0 ± 102.3 | 0.262 |
| Infratemporal, 30°–60° | 329.4 ± 69.2 | 335.4 ± 174.0 | 0.538 |
| Infratemporal, 60°–100° | 238.4 ± 50.6 | 234.9 ± 102.4 | 0.342 |
| Supranasal, 30°–60° | 269.2 ± 64.7 | 291.1 ± 97.3 | 0.837 |
| Supranasal, 60°–100° | 218.9 ± 56.5 | 241.2 ± 92.7 | 0.902 |
| Infranasal, 30°–60° | 233.6 ± 64.3 | 269.1 ± 176.5 | 0.837 |
| Infranasal, 60°–100° | 168.1 ± 40.0 | 192.9 ± 76.4 | 0.902 |

The data are shown as means with standard deviations. P-values were calculated using the Mann–Whitney U test.
